# Physical activity, post-traumatic stress disorder, and exposure to torture among asylum seekers in Sweden: a cross-sectional study

**DOI:** 10.1186/s12888-021-03461-2

**Published:** 2021-09-16

**Authors:** Henrik Nilsson, Catharina Gustavsson, Maria Gottvall, Fredrik Saboonchi

**Affiliations:** 1 Department of Health Sciences, The Swedish Red Cross University College, PO Box 1059, SE-141 21 Huddinge, Sweden; 2 Division of Insurance Medicine, Department of Clinical Neuroscience, Karolinska Institutet, SE-171 77 Stockholm, Sweden; 3 The Swedish Red Cross Treatment Center for Persons Affected by War and Torture, PO Box 166, SE-201 21 Malmö, Sweden; 4 Center for Clinical Research Dalarna, Uppsala University, Nissers väg 3, SE-791 82 Falun, Sweden; 5 Clinical Psychology in Health Care, Department of Women’s and Children’s Health, Uppsala University, SE-751 85 Uppsala, Sweden

**Keywords:** Physical activity, Asylum seeker, Refugee, Trauma, Torture, Post-traumatic stress disorder, PTSD, Post-migration stress, Mental health

## Abstract

**Background:**

Forced migrant populations have high rates of trauma-related ill health, including post-traumatic stress disorder (PTSD). Physical activity (PA) is well-established as an effective stress reliever, while insufficient PA is associated with adverse effects on both mental and physical health. The aim of this study was to examine the prevalence of different levels of PA and its association with PTSD symptom severity, controlled for exposure to torture, among asylum seekers in Sweden.

**Methods:**

A cross-sectional survey study, with data from 455 asylum seekers, originating from Afghanistan, Eritrea, Iraq, Somalia, and Syria, residing at large housing facilities across Sweden. Level of PA was assessed by the Exercise Vital Sign and categorized as; Inactive, Insufficient PA, and Sufficient PA. Prevalence estimates for proportions of different levels of PA were calculated. Analysis of variance were conducted to determine the association between levels of PA and PTSD symptom severity, measured by the Harvard Trauma Questionnaire. Multivariable logistic regression analysis was performed to determine the contribution of PA on PTSD beyond sex, age, and exposure to torture.

**Results:**

About half of the participants (53.3, 95% CI: 48.6–58.1) met the recommendations for Sufficient PA. One third of the participants (33.3, 95% CI: 28.7–37.8) were insufficiently engaged in PA, and 13.4% (95% CI: 10.1–16.7) were inactive. There was a significant difference in PTSD symptom severity between groups of asylum seekers with different levels of PA (*F*_(2, 316)_ = 23.15, *p* < .001). When controlling for sex, age, and exposure to torture, Sufficient PA was found to be associated with less PTSD symptom severity compared to both Insufficient PA (*B* = 0.297, *SE* = 0.086, *p* < .001) and Inactive (*B* = 0.789, *SE* = 0.104, *p* < .001).

**Conclusions:**

Insufficient PA was common among the asylum seekers and our findings suggest that more PA is highly associated with lower PTSD symptom severity. An increased focus on assessment and promotion of PA is justified and discussed as particularly pertinent considering the much extended time of asylum-seeking processes. The results support previous evidence of PA as a potentially important factor in the context of PTSD and forced migrants’ health.

## Background

As a result of war, conflict, persecution and human rights violations, the numbers of forcibly displaced people have escalated in recent years. Almost 80 million people are currently being forced to flee their homes, whereof 26 million are refugees and 4.2 million are asylum seekers, i.e., seeking international protection but whose claim for refugee status has not yet been determined [1]. Sweden is one of the largest European recipients of refugees and asylum seekers, and has received more than 500,000 applications for asylum over the past decade [2].

Forced migrant populations are subjected to extreme stress related to both ongoing living difficulties [3–7] and high rates of pre-migratory exposure to trauma [7–9], e.g., war at close quarters, witnessing the death of others, generalized violence, and torture. Experiences of interpersonal trauma, and especially torture, are powerful predictors of mental ill health in general and symptoms of post-traumatic stress disorder (PTSD) in particular [9–11]. Torture is by definition a grave violation of human rights. It is prohibited in international human rights law, but still practiced in over 140 countries, including Syria, Afghanistan, Somalia, and other countries where many refugees originate [12]. Furthermore, exposure to trauma and extreme stress often continue both during and after the migration. Experiences such as separation from family, dangerous travel methods, continued conflicts in home country, prolonged and uncertain asylum processes, socioeconomic difficulties, and perceived racism may add to previous traumatic experiences and contribute to the development or maintenance of both mental and physical health problems, including symptoms of PTSD [3–6, 10, 13, 14]. Consequently, refugees and asylum seekers are at high risk for complex health conditions. Previous research has demonstrated high rates of both PTSD and associated comorbidity such as depression and anxiety [4, 7, 9, 13], sleep disturbances, chronic pain, fatigue, and functional impairments [14–18]. In all, these complex health issues constitute substantial health care challenges [10, 19, 20] and a significant public health problem in receiving countries [19, 21].

PTSD and other stress-related disorders are also associated with poor health behaviors, including low levels of physical activity (PA) [22–25], which may additionally complicate the already burdened health situation among forced migrant populations. The health benefits of PA are well established [26] and regular PA is known as an effective stress reliever and associated with improved psychological wellbeing [27]. Both international [28] and national PA guidelines [29] suggest that at least 150 min of moderate-intensity PA per week is needed to obtain important health benefits. Conversely, insufficient PA (i.e. < 150 min per week) is strongly connected to adverse effects on both mental and physical health, including increased risks of chronic medical conditions such as diabetes, hypertension, and cardiovascular disease [30], which forced migrant populations are already at heightened risk for [18, 31]. Previous research on PA and PTSD has shown a potentially complex interrelationship, including different suggestions of directionality, and indications that low levels of PA may act as a reciprocal maintaining factor of both PTSD and comorbidity [23–25, 32, 33]. For example, insufficient PA is generally proposed to be bi-directionally associated with depression, sleeping problems, and chronic pain, while these symptoms or conditions are also associated with symptoms of PTSD [14, 16, 34, 35]. Interventions including PA and exercise have been shown to alleviate the severity of PTSD symptoms and to have beneficial effects on prevalent psychological comorbidity such as depression and anxiety [36–38]. Further, regular PA is also associated with a range of favorable health aspects that may be of particular importance concerning forced migrants’ health, including increased energy and daily life functioning, resilience and self-management of stress, sleep quality, physical health status, self-esteem and self-confidence, cognitive performance, and improved social relations [26, 27, 39, 40]. However, the potential impact of PA has received scarce research attention in the field of PTSD and forced migrants’ health, and particularly among the vulnerable group of asylum seekers. Despite the complex mental health needs related to stress and trauma exposure; neither the prevalence of PA, nor its association to PTSD and exposure to grave trauma, especially torture, has to our knowledge previously been examined.

Thereby, the aim of this study was to (i) assess the prevalence of PA among a cohort of asylum seekers in Sweden, in total and stratified by sex and age group, (ii) examine the differences in PTSD symptom severity between asylum seekers with different levels of PA, and (iii) examine the associations between PA and PTSD, controlled for exposure to torture.

## Methods

### Study design

A cross-sectional survey design was used for the study. The reporting adheres to the STROBE guidelines for reporting observational studies [41].

### Participants and setting

The study setting was three large housing facilities for asylum seekers located in Sweden. Inclusion criteria for participation in the study were: having an asylum seeker status, belonging to one of the largest refugee groups at present in Sweden (Afghanistan, Eritrea, Iraq, Somalia, or Syria), and being at least 18 years old. The total number of eligible participants was 1698. Data on PTSD and exposure to torture among the sample has been published previously [6].

### Data collection and procedure

Data collection for this study was part of a comprehensive survey to asylum seekers in Sweden and was conducted from May 2016 to March 2018 [6]. A questionnaire including sociodemographic data, trauma history, measures of mental health, and level of PA, was used. Translations of the questionnaire were carried out by certified translators and reviewed by other certified translators or by bilingual staff members of the Swedish Red Cross University College. The Swedish Migration Board provided a list of eligible participants, including sociodemographic data, based on the ethical approval from the Regional Ethical Review Board. Written and oral information about the study was provided to eligible participants in their native language (Arabic, Dari, or Tigrinya), including that completing and returning the questionnaire would be considered informed consent. Each participant was then assigned a unique ID number and all further data handling was performed on anonymized dataset. At each housing facility, volunteers and Red Cross staff members distributed the questionnaires and the written information to eligible participants. At each site, professional interpreters were also available during data collection. All completed questionnaires were scanned and compiled for analysis.

### Measures

#### Sociodemographic characteristics

Data on sex, age, country of origin, and year of immigration was provided by the Swedish Migration Board. Age of participants was categorized into two groups: 18–30 years and 31–64 years. Additional sociodemographic data, including highest educational level and family situation, were collected by self-report from the participants.

#### Physical activity

Level of PA was assessed by the Exercise Vital Sign (EVS) [42]. The EVS is a brief PA questionnaire that was designed to identify individuals who are not meeting PA recommendations. It was selected by application of the “decision matrix” in the *Guide to the Assessment of Physical Activity: Clinical and Research Applications* [43]. According to these guidelines, the EVS is a commonly used global PA questionnaire that may be used to identify PA guidelines and/or to provide a classification. It assesses the average time spent in moderate to strenuous activity, displayed in minutes per week, by multiplying the responses on two self-report questions: 1) “*On average, how many days per week do you engage in moderate to strenuous exercise (like a brisk walk)?*” (response options: 1–7 days) and 2) “*On average, how many minutes per day do you engage in exercise at this level?*” (response options for minutes per day: 0, 10, 20, 30, 40, 50, 60, 90, 120, and ‘150 or more’). In this study, the EVS score was divided into three PA categories according to established cut-offs in PA guidelines [28]: Inactive (0 min·wk.^−1^), Insufficient PA (1–149 min·wk.^−1^), and Sufficient PA (≥ 150 min·wk.^−1^). The questionnaire has shown good discriminant validity being administrated to more than 1.5 million individuals in a US healthcare setting and by using the same classification as in this study [42]. The EVS has also been recently evaluated against accelerometry in an ethnically/racially diverse sample, providing preliminary evidence of acceptable validity and high test-retest reliability [44].

#### Post-traumatic stress disorder

To assess symptoms of PTSD, the trauma symptom scale (part IV) of the Harvard Trauma Questionnaire (HTQ) [45] was used. The HTQ symptom scale is cross-culturally validated and frequently used among refugee populations for both evaluation of treatment outcome [46] and in screening for PTSD [10, 13]. The scale consists of 16 PTSD symptom items, corresponding to the Diagnostic and Statistical Manual of Mental Disorders, 4th edition, (DSM-IV) criteria for PTSD. The PTSD symptoms is assessed by asking respondents to rate how much they have been bothered by each of the 16 symptoms during the past week on a 4-point scale: *not at all (1)*, *a little bit (2)*, *quite a bit (3)*, or *extremely (4)*. PTSD symptom severity is computed by averaging the responses on the list of symptoms, giving a score between 1 and 4. Higher score indicates more symptom severity. In this study, a mean item score of ≥2.06 was used to define positive PTSD cases, based on previously established value of PTSD among primary care patients in Bosnia Herzegovina [47], and previously used among refugees in Sweden [7, 11]. According to a recent study by Vindbjerg et al. [48], the HTQ may also be divided into two subscales: a 9-item arousal/intrusion subscale (AIS) and a 7-item avoidance/numbing subscale (ANS). In the present study, these subscales were used to examine differences between clusters of PTSD symptoms in relation to different levels of PA.

#### Exposure to torture

Two questions from the Refugee Trauma History Checklist (RTHC) [49] was used to assess whether participants had experienced torture before and/or during their flight. In addition to the questions regarding torture exposure, the checklist includes questions targeting other potentially traumatic events, e.g., war at close quarters, violence, and forced separation from family and close friends. All questions are answered on a binary outcome scale (Yes/No). The two questions regarding exposure to torture prior to flight and during the flight were combined to establish exposure, i.e., participants who endorsed either or both of the questions were considered exposed. The checklist has been used in previous studies among refugee populations in Sweden [7, 11].

### Statistical analysis

Descriptive statistics was used to describe participant characteristics. Prevalence estimates and their corresponding 95% confidence intervals (CI) were calculated to outline the proportions for each EVS category both within the total sample and stratified by sex and age group. Although no significant testing of the differences between strata were performed, non-overlapping CI’s were viewed as indicating significant differences in prevalence estimates.

The differences between participants designated to different EVS categories (levels of PA) in regard to HTQ score (PTSD symptom severity) and its subscales, AIS and ANS, were assessed by univariate and multivariate Analysis of Variance (ANOVA and MANOVA). One-way ANOVA for independent group was used to assess the difference in regard to total HTQ score, while MANOVA was used for assessing the overall differences in AIS and ANS due to high intercorrelation between these symptom clusters (*r* = 0.83). Upon establishing an overall significance indicated by *Wald* and *F* statistics, follow-up univariate ANOVAs were then used to explore the differences in AIS and ANS separately. Pairwise comparisons between each pair of EVS categories were done by Hochberg’s GT2 test in order to account for different samples sizes in each category, and Dunnett’s T3 test in order to account for possible heterogeneity of variance. In all analyses, the significance level was set to *p* ≤ .05.

Assumption about homogeneity of variance for ANOVA for total HTQ score, and MANOVA for ANS and AIS, were tested by Levene’s Test and Box’s Test of Equality of Covariance Matrices. Neither of the tests were significant. Furthermore, normal distribution of residual for both HTQ total score and AIS and ANS were examined and showed neither substantive departure of normality nor presence of outliers. Although the assumptions for ANOVA and MANOVA were confirmed, the analyses were also performed by bootstrapping the CIs as a further mean of guarding against violations of assumptions.

Multiple regression analysis with HTQ score as the dependent variable was performed to assess the overall associations between sex, age, exposure to torture, and level of PA, indicated by EVS categories. Stepwise hierarchical regression in three steps were applied in order to assess the contribution of the EVS categories to PTSD symptom severity, indicated by HTQ score, by controlling for sex, age, and exposure to torture. Subsequently, the variables sex and age group were entered in the first step of the model, exposure to torture was added in the second step, and lastly, the variable EVS categories were added in the final step of the model. Analyses were performed by bootstrapping in order to provide robust bootstrap adjusted standard errors of the estimates and to guard against the violation of assumptions of normality. All statistical analysis were conducted using the IBM SPSS Statistics for Windows, version 24.0.

## Results

### Characteristics of participants

In total, 455 asylum seekers were included in the study by responding to the questionnaire (26.8% of the eligible residents). Characteristics of the participants are presented in Table 1. In brief, the majority of participants were men, between 18 and 30 years of age, not living with a partner, and had 9 years or less of education. About one third originated from Afghanistan, one third were from Syria, and the remaining from Somalia, Eritrea, and Iraq. Among the participants, 56% had been exposed to torture (57% among women and 55% among men). Among the participants who responded to the HTQ (*n* = 319), 61% reported PTSD symptom severity above or equivalent to the recommended cut-off (≥ 2.06) to define a checklist-positive diagnosis. The mean PTSD symptom severity score was 2.28 (*SD* = 0.771).

**Table 1:** Sociodemographic characteristics, including exposure to torture and PTSD, among the participants (*N* = 455)

Sample characteristics	n	%
**Sex**		
Men	333	73.2
Women	122	26.8
**Age groups**		
18–30 years	269	59.1
31–64 years	186	40.9
**Educational level**		
≤ 9 years	261	57.4
9–12 years	101	22.2
> 12 years	74	16.3
*Missing*	19	4.2
**Family situation**		
Living with a partner	119	26.2
Not living with a partner	261	57.4
Divorced/widow	37	8.1
*Missing*	38	8.4
**Year of immigration**		
Prior to 2014	20	4.4
2014	33	7.3
2015	362	79.6
2016	15	3.3
2017	20	4.4
2018	4	0.9
*Missing*	1	0.2
**Country of origin**		
Afghanistan	154	33.8
Eritrea	45	9.9
Iraq	38	8.4
Somalia	64	14.1
Syria	145	31.9
Stateless	9	2.0
**Torture**		
Exposure to torture before and/or during migration	254	55.8
*Missing*	25	5.4
**PTSD**^a^		
PTSD according to HTQ cut-off ≥2.06	194	60.8
*Missing*	136	29.9

a Percentage of positive PTSD cases are based on those responding to the HTQ, n = 319

PTSD, post-traumatic stress disorder; HTQ, Harvard Trauma Questionnaire; N, population size; n, sample size

### Prevalence of physical activity

The level of PA, in total as well as stratified by sex and age group, is presented in Table 2. About half of the participants (53.3, 95% CI: 48.6–58.1) met the recommendations for a sufficient level of PA. One third of the participants (33.3, 95% CI: 28.7–37.8) were insufficiently engaged in PA, and additionally 13.4% (95% CI: 10.1–16.7) were inactive.

**Table 2:** Prevalence of physical activity categorized as Inactive, Insufficient PA, and Sufficient PA

	Inactive % (95% CI)	Insufficient PA % (95% CI)	Sufficient PA % (95% CI)
Total sample (*n* = 418)	13.4 (10.1–16.7)	33.3 (28.7–37.8)	53.3 (48.6–58.1)
18–30 years (*n* = 244)	13.5 (9.2–17.8)	31.6 (25.7–37.4)	54.9 (48.7–61.2)
31–64 years (*n* = 174)	13.2 (8.2–18.3)	35.6 (28.5–42.7)	51.1 (43.7–58.6)
Women (total, *n* = 115)	19.1 (11.9–26.3)	35.7 (26.9–44.4)	45.2 (36.1–54.3)
18–30 years (*n* = 59)	23.7 (12.9–34.6)	30.5 (18.8–42.3)	45.8 (33.1–58.5)
31–64 years (*n* = 56)	14.3 (5.1–23.5)	41.1 (28.2–54.0)	44.6 (31.6–57.7)
Men (total, *n* = 303)	11.2 (7.7–14.8)	32.3 (27.1–37.6)	56.4 (50.9–62.0)
18–30 years (*n* = 185)	10.3 (5.9–14.6)	31.9 (25.2–38.6)	57.8 (50.7–65.0)
31–64 years (*n* = 118)	12.7 (6.7–18.7)	33.1 (24.6–41.5)	54.2 (45.2–63.2)

Levels of PA are based on the EVS total score of minutes per week in moderate to strenuous exercise; Inactive (0 min·wk.^−1^), Insufficient PA (1–149 min·wk.^−1^), and Sufficient PA (≥ 150 min·wk.^−1^)

PA, physical activity; EVS, Exercise Vital Sign; CI, confidence interval; n, sample size

The analyses indicated lower level of PA among women than men, although CIs were more or less overlapping. A possible difference between women and men was found among those inactive and of younger age (18–30 years), for whom the estimates were 23.7% for women and 10.3% for men, but also here the CIs were overlapping. No differences in prevalence of PA were detected between age groups for the total population. Due to skewed distribution and too small strata, stratified estimates on basis of country of origin could not be reliably provided.

### Differences in PTSD symptom severity between groups of asylum seekers with different levels of physical activity

Table 3 shows that there was a significant difference in PTSD symptom severity between groups of asylum seekers with different levels of PA (*F*_(2, 316)_ = 23.15, *p* < .001). Pairwise post-hoc tests showed a clear pattern of associations, i.e., the Sufficient PA group had less PTSD symptom severity compared to both the Insufficient PA group (*p* = .004) and the Inactive group (*p* < .001), and the Insufficient PA group had less PTSD symptom severity compared to the Inactive group (*p* < .001).

**Table 3:** Differences in PTSD symptom severity between groups of asylum seekers with different levels of physical activity

	Inactive *n* = 42 M (SD)	Insufficient PA *n* = 108 M (SD)	Sufficient PA *n* = 169 M (SD)	*F*_(2, 316)_	*p*
HTQ total (PTSD symptom severity)	2.89 (0.598)	2.37 (0.756)	2.07 (0.727)	23.15	< .001
AIS subscale	2.95 (0.626)	2.39 (0.801)	2.11 (0.803)	20.06	< .001
ANS subscale	2.81 (0.659)	2.33 (0.766)	2.00 (0.731)	22.07	< .001

Levels of PA are based on the EVS total score of minutes per week in moderate to strenuous exercise; Inactive (0 min·wk.^−1^), Insufficient PA (1–149 min·wk.^−1^), and Sufficient PA (≥ 150 min·wk.^−1^)

PTSD symptom severity is based on the HTQ total score and divided into the AIS and ANS subscales

PTSD, post-traumatic stress disorder; PA, physical activity; HTQ, Harvard Trauma Questionnaire; AIS, arousal and intrusion symptom subscale; ANS, avoidance and numbing symptom subscale; EVS, Exersice Vital Sign; n, sample size; *M*, mean; *SD*, standard deviation; *F*, Fishers test; *p*, probability value

In regard to symptom clusters of PTSD, as indicated by the AIS and ANS, MANOVA revealed overall significant differences in symptom severity between groups with different levels of PA (Wilk’s ˄ = 0.868, *F*_(4, 630)_ = 11.58, *p* < .001). Follow-up univariate ANOVA established differences in regard to both the AIS (*F*_(2, 316)_ = 20.06, *p* < .001) and the ANS (*F*_(2, 316)_ = 22.07, *p* < .001). Pairwise post-hoc tests showed a similar pattern of associations for the AIS and ANS as with the HTQ in total, i.e., the Sufficient PA group had less symptom severity compared to both other PA groups, and the Insufficient PA group had less symptom severity compared to the Inactive group (all significance tests between *p* < .001 and *p* = .016).

### Associations between physical activity, exposure to torture, and severity of PTSD symptoms

As shown in Table 4, the first model in the regression analysis revealed that being a woman was associated with less PTSD symptom severity in comparison with being a man (*B* = − 0.207, *SE* = 0.001, *p* < .038). No significant association was found between age groups and symptom severity. This model accounted for 2% of the total variance in PTSD symptom severity.

**Table 4:** Associations between PTSD symptom severity and sex, age group, exposure to torture, and level of physical activity

Covariates	Model 1^a^				Model 2^b^				Model 3^c^			
	*B*	*SE B*	95% CI	*p*	*B*	*SE B*	95% CI	*p*	*B*	*SE B*	95% CI	*p*
Sex												
Woman	−0.207	0.100	−0.416 - -0.023	.038	−0.259	0.096	−0.468 --0.081	.010	−0.321	0.087	−0.501 --0.161	.002
Man	Ref.				Ref.				Ref.			
Age group												
18–30	Ref.				Ref.				Ref.			
31–64	−0.047	0.088	−0.213 -0.127	.607	−0.015	0.085	−0.167 -0.166	.869	−0.009	0.079	−0.155 -0.149	.905
Exposure to torture												
Yes					0.526	0.085	0.358–0.700	< .001	0.469	0.083	0.308–0.631	< .001
No					Ref.				Ref.			
Level of PA												
Inactive									0.789	0.104	0.591–1.005	< .001
Insufficient PA									0.297	0.086	0.133–0.465	< .001
Sufficient PA									Ref.			
*R*^*2*^	0.02				0.13				0.25			

a Model 1 includes the variables sex and age

b Model 2 includes the variables sex, age, and exposure to torture

c Model 3 includes the variables sex, age, exposure to torture, and PA level

*SE*, *p,* and CI are based on 1000 bootstrap samples

PTSD symptom severity is based on the HTQ total score

Levels of PA are based on the EVS total score of minutes per week in moderate to strenuous exercise; Inactive (0 min·wk.^-^^1^), Insufficient PA (1–149 min·wk.^-^^1^), and Sufficient PA (≥ 150 min·wk.^-^^1^)

PTSD, post-traumatic stress disorder; PA, physical activity; HTQ, Harvard Trauma Questionnaire; EVS, Exercise Vital Sign; *B*, unstandardized beta; *SE*, standard error; CI, confidence interval; *p*, probability value; *R*^*2*^, coefficient of determination

In the second model, which in addition to sex and age group also included exposure to torture, associations were found between PTSD symptom severity and sex and exposure to torture respectively. Being exposed to torture was associated with significantly higher PTSD symptom severity compared to not being exposed to torture (*B* = 0.526, *SE* = 0.085, *p* < .001). The addition of exposure to torture in this model accounted for an additional 11% of the total variance in PTSD symptom severity and the change in *R*^*2*^ was significant (*F*_(1, 309)_ = 40.51, *p* < .001).

In the final model, all the above-mentioned variables were included with the addition of level of PA. All the above associations persisted, and Sufficient PA was found to be negatively associated with PTSD symptom severity. Both Insufficient PA (*B* = 0.297, *SE* = 0.086, *p* < .001) and Inactive (*B* = 0.789, *SE* = 0.104, *p* < .001) was significantly associated with more symptom severity. This model accounted for 25% of the total variance in PTSD symptom severity, i.e., the addition of the variable PA uniquely explained an additional 12% of the total variance in PTSD symptom severity. This change in *R*^*2*^ was also significant (*F*_(2, 307)_ = 23.39, *p* < .001).

## Discussion

Despite the well-known impact of PA on mental health and wellbeing [26–28], little is currently known about PA among asylum seekers, a population which is known to display high prevalence of trauma exposure and mental ill health including PTSD [3, 4, 8, 9]. The results of this study revealed several noteworthy findings. First, almost 50% of the study population did not meet the international recommendations for a sufficient level of health-promoting PA, and were classified as either inactive or insufficiently engaged in PA. Second, both inactive and insufficient PA were found to be significantly associated with more PTSD symptom severity compared to those who met the recommendations for a sufficient level of PA. Finally, this association persisted and additionally accounted for a marked proportion of the variance in PTSD symptom severity even when analyses were controlled for sex, age, and exposure to torture.

A proportion corresponding to almost half of the cohort of this study not meeting the recommendations of sufficient PA appears as noticeably high compared to both international and national estimates of insufficient PA (also including those being completely inactive). According to the WHO Global Health Observatory data of 2016 [50], the worldwide estimates of insufficient PA were in average 27.5% (31.7% among women and 23.4% among men), whereas the same estimate for the general population in Sweden was 23.1% (24.7% among women and 21.5% among men). Our findings of possible differences between women and men are in line with these data as well as previous research reporting female gender to be associated with higher levels of insufficient PA across most countries and populations [51, 52]. However, the disparities between sexes varies considerably within and between countries. Differences in both level and types of PA has been suggested to be highly influenced by social and cultural norms and practices [52, 53], whereby some cultural norms have been recognized as particularly discouraging of women’s participation in PA, such as conservative dress codes, lack of social and community support, and lack of gender segregated facilities [54]. There is also a wide variation of overall country specific estimates of insufficient PA across both cultural and geographical regions [51], which is believed to be explained by numerous factors at multiple levels, including demographical, psychosocial, sociocultural, and environmental variables [52]. However, potential differences on basis of country by origin could not be reliable provided in this study, and neither could our overall estimates be further examined against relevant national estimates since such data are not available for either Afghanistan, Syria, or Somalia (i.e., representing almost 80% of our study population).

In general, the estimates of insufficient PA are higher in high-income countries than in low-income countries, with in average 36.8% of the populations in high-income countries being insufficiently active compared to 16.2% in low-income countries [51]. Most asylum seekers originate from low- or middle-income countries [1], indicating that the high prevalence of insufficient PA in our study may possibly be viewed as attributed to overall conditions associated with the process of forced migration, trauma exposure, and being an asylum seeker. More specifically, previous research has established that people with severe mental ill health are less likely to engage in PA and are more sedentary in comparison to the general population [55–57], and, the process of cultural change and acculturation has been suggested to additionally influence almost all other correlates of PA, including key variables such as social inequalities, social support, and motivation [53, 58]. Given the high rates of trauma-related ill health [3, 4], disrupted daily life and work routines from home country [4, 6], as well as other barriers to engage in PA that are likely to be faced by forced migrant populations, such as economic strain, access to facilities, language difficulties, unfamiliarity with the environment, loss of social network, and lack of motivation when living under extreme stress and uncertainty about the future [53, 57–59], it may be concluded that asylum seekers are more readily susceptible to insufficient PA. An increased focus on assessment and promotion of PA is thereby justified. This may be seen as particularly pertinent considering the currently much extended time of the asylum-seeking processes in many host countries [1]. In order for such actions to be efficient, particular attention to contextual and cultural factors may be necessary.

The issue of further assessment and promotion of PA among asylum seekers may also be seen in light of the *WHO Global Action Plan on Physical Activity 2018–2030* [60], including an accentuated need to identify high risk groups of insufficient PA, to increase the knowledge and delivery of context and culturally sensitive actions to promote PA, and subsequently, to facilitate both mental and physical health. According to the few recent reports on PA and sport among forced migrant populations, such actions may not only be attributed to physical and psychological outcomes but also as a facilitator of social health, such as greater acculturation, integration, social inclusion, and feelings of belonging [38, 58, 61–63]. In regard to promotion of PA, and partially related to our findings of more than one in eight asylum seekers being completely inactive, it may also be noted that recent research has demonstrated important health benefits even at much lower doses than advocated by generic PA guidelines, and especially when moving from completely inactive to some activity [64, 65]. Further, as it has been recognized that the current threshold of 150 min per week may seem unattainable or even discoursing for some people, it has also been argued that the promotion of any engagement in PA may, in some cases, be advisable and which has been particularly pronounced as a potentially important message among people currently inactive and/or suffering from severe mental ill health [65, 66].

Our findings of a clear pattern of differences in PTSD symptom severity relating to level of PA, support that there is an association between mental ill health and insufficient PA. Previous research has found substantial reductions in PA and active leisure time habits after the onset of PTSD [25], which may indicate a direction of mental illness as a contributing factor or an antecedent to insufficient PA. However, research has also shown that low levels of PA can act as a major risk factor for the development and maintenance of mental ill health, including PTSD and comorbidity [33, 35, 56, 67]. It is thus possible that a similar pattern is reflected in the findings of the present study. In that case, low levels of PA may adversely influence mental health and PTSD symptom severity among asylum seekers who have been exposed to severe traumatic experiences. Taken together, the associations indicated by the differences in regard to insufficient PA and PTSD symptom severity may as well be bi-directional in the same line as delineated by the Mutual Maintenance Model [68]. This model proposes that PTSD symptoms and chronic pain are mutually maintaining conditions, and that there may be several pathways by which both conditions can lead to an escalation of symptoms and distress following trauma. Concerning PA, presuming an equivalent analogy, this would imply that PTSD symptom severity may partially influence level of PA while also simultaneously be adversely influenced by insufficient PA. Promotion of PA may, in this case, be seen as both a preventative measure (i.e., to reduce the risks of maintenance or escalation of PTSD symptoms and PA-related comorbidity) and an attempt to alleviate current symptom severity. Based on this hypothetical analogy with the Mutual Maintenance Model, it should, however, also be noted that the mediating mechanisms between PTSD and chronic pain may be very different, and that chronic pain may in itself have an essential role in the links between PA and PTSD. For example, there are several proposed symptom overlaps between pain and PTSD, such as anxiety, avoidance behavior, and elevated somatic focus, which may also influence level of PA. Further, our findings of differences in PTSD symptom severity also between those being completely inactive compared to those with insufficient PA, might yield some support to previous suggestions that even a low dose of PA may be associated with important aspects of health [64, 65]. However, on basis of our results, the direction between respective associations remains unclear.

The possibility that levels of PA may, to some extent, influence PTSD symptom severity, was furthermore supported by the results of the analyses in which exposure to torture, as an established main predictor of PTSD [9, 10], was controlled for. While exposure to torture displayed an expected high explanatory function for PTSD symptom severity, insufficient PA provided additional high explanation for the variation in PTSD symptom severity beyond exposure to torture. Although those not exposed to torture may still have experienced other severe trauma, the overall pattern indicates that insufficient PA may act as a risk factor, mediator, or aggravator of PTSD symptom severity for those inflicted by severe trauma. On the other hand, and in line with the analogy with the Mutual Maintenance Model, higher symptom severity may also act as a risk factor for or mediator of lower PA. Nevertheless, as the cross-sectional nature of our data precludes causal inferences, the results need to be replicated by means of longitudinal studies in order to clarify causality and to assess each factor’s contribution to symptom severity. In addition, there may be other symptoms or conditions that may influence both PTSD symptoms and level of PA, such as poor social support [69], low self-efficacy [70], and as previously highlighted, chronic pain and sleeping problems [15–17], which are common in the context of displacement and exposure to severe trauma, and thus, warranted further investigation in future studies.

Our results regarding different clusters of PTSD symptoms, i.e., arousal/intrusion and avoidance/numbing, showed similar patterns of differences and associations with insufficient PA as that of the overall PTSD symptom severity. These results deviate to some extent from the inferences of a systematic review by Vancampfort et al. [32], suggesting that the only correlate consistently associated with low PA in people with PTSD is symptoms of hyperarousal. Our findings, however, could also be viewed in light of other studies that have suggested that physical and social inactivity may also comprise a part of avoidance symptoms and negative cognitions and mood, e.g., avoidance of trauma-related stimuli, feeling isolated, and decreased interest in activities [25, 35, 37, 71]. Moreover, these symptom clusters may as well be closely interrelated in regard to their influence on PA, such as avoiding activities or exercise due to lack of energy or motivation, fear of bodily arousal (e.g., muscle tension, increased heart rate, shortness of breath), or fear of intrusive memories that may be triggered by physical strain. In addition, despite a recently increased attention [38, 40], the role of social, cultural, environmental, and policy factors on PA participation among people with PTSD in general [32], and forced migrant populations in particular [53, 58, 59], is still understudied and need to be further addressed by future research.

### Strengths and limitations

To our knowledge, this is the first assessment of prevalence of PA and its association to PTSD in a cohort of asylum seekers in a high-income country setting. The use of a cross-culturally validated measure for PTSD and well-established measures of PA, availability of information on the sample population, and an adequate sample size that provided necessary statistical power for assessment of associations and the possibility to establish the actual response rates compared to the total eligible study population, are the strengths of the study. It is, however, a limitation to the study that the proportion of those choosing to participate was just slightly more than one fourth of the total population. Still, such response rate is common in surveys conducted among hard-to-reach populations in general, and in forced migrant populations in particular. Thus, obtaining data on PA and severity of PTSD symptoms among 26.8% of all eligible individuals could be considered acceptable in this context. However, the generalizability of the estimates of prevalence of PA to other settings and other forced migrant groups may be limited.

Further, it has been reported that mental health problems may be more common among non-respondents [72, 73], which may also bias the results concerning the PA prevalence estimates. However, since the estimates of associations, compared to that of population characteristics, are less prone to bias caused by non-response [73, 74], the results concerning associations of PA with PTSD symptom severity may be viewed as less influenced by this condition. Our findings regarding PA prevalence should also be interpreted with caution due to the subjective nature of assessment by the EVS. Specifically, it has previously been reported that many self-report PA questionnaires, including the EVS, generally overestimate the minutes of PA per week compared to objective measures such as accelerometry or direct observations [44, 75]. It is thus possible that the true proportion of insufficiently active asylum seekers may be even higher than suggested by our results.

The selection of torture as a single worst trauma may be another limitation, as it has been suggested that a cumulative trauma score may provide more explanatory power. However, since the RTHC only includes eight broad categories of potentially traumatic events which differ in terms of both specificity and severity of trauma, a valid a cumulative score for this study could not be estimated on basis of the available data.

Moreover, given the cross-sectional design and the observational data in the study, causal directions in links between PTSD symptomology and PA cannot be established by means of the obtained empirical data. Bearing this in mind, our ambition has not been to assess the causality of these associations and we have opted to discuss the possible directions of these associations against the background of the existing literature. Nevertheless, the results provide some evidence for the potential importance of PA in regard to PTSD symptomatology and mental health of asylum seekers. Our results also encourage more in-depth examination of PA and mental health among forced migrants and provide an interesting starting point for future studies using prospective and longitudinal designs.

## Conclusions

Insufficient PA appeared to be more prevalent among the cohort of asylum seekers in this study compared to the reported prevalence in general populations and our findings suggest that more PA is highly associated with lower PTSD symptom severity. An increased focus on assessment and promotion of PA among the trauma-prone populations of asylum seekers is thereby justified, and further discussed as particularly pertinent considering the currently much extended time of asylum-seeking processes. The results of this study support previous evidence of PA as a potentially important factor in the context of PTSD and forced migrants’ health, however, further research is warranted to clarify causality and to examine the potential efficacy of PA promotion in this regard.

## Data Availability

The datasets used and analysed during the current study are available from the corresponding author on reasonable request.

## Abbreviations

AIS: arousal and intrusion subscale
ANS: avoidance and numbing subscale
DSM-IV: Diagnostic and Statistical Manual of Mental Disorders, 4th edition
EVS: Exercise Vital Sign
HTQ: Harvard Trauma Questionnaire
PA: physical activity
PTSD: post-traumatic stress disorder
RTHC: The Refugee Trauma History Checklist

## References

[1] United Nations High Commissioner for Refugees (UNHCR): Figures at a glance. https://www.unhcr.org/figures-at-a-glance.html (2020). Accessed 18 December 2020.

[2] Swedish Migration Agency: Statistics. https://www.migrationsverket.se/English/About-the-Migration-Agency/Statistics/Asylum.html (2020). Accessed 18 December 2020.

[3] Li SSY, Liddell BJ, Nickerson A (2016). The relationship between post-migration stress and psychological disorders in refugees and asylum seekers. Current psychiatry reports.

[4] Laban CJ, Gernaat HBPE, Komproe IH, Van Der Tweel I, De Jong JTVM (2005). Postmigration living problems and common psychiatric disorders in Iraqi asylum seekers in the Netherlands. J Nerv Ment Dis.

[5] Lindencrona F, Ekblad S, Hauff E (2008). Mental health of recently resettled refugees from the Middle East in Sweden: the impact of pre-resettlement trauma, resettlement stress and capacity to handle stress. Soc Psychiatry Psychiatr Epidemiol.

[6] Solberg Ø, Vaez M, Johnson-Singh CM, Saboonchi F (2020). Asylum-seekers' psychosocial situation: a diathesis for post-migratory stress and mental health disorders?. J Psychosom Res.

[7] Tinghög P, Malm A, Arwidson C, Sigvardsdotter E, Lundin A, Saboonchi F (2017). Prevalence of mental ill health, traumas and postmigration stress among refugees from Syria resettled in Sweden after 2011: a population-based survey. BMJ Open.

[8] Sigvardsdotter E, Vaez M, Rydholm Hedman A-M, Saboonchi F (2016). Prevalence of torture and other warrelated traumatic events in forced migrants: a systematic review. Torture : quarterly journal on rehabilitation of torture victims and prevention of torture.

[9] Steel Z, Chey T, Silove D, Marnane C, Bryant RA, van Ommeren M (2009). Association of Torture and Other Potentially Traumatic Events with Mental Health Outcomes among Populations Exposed to mass conflict and displacement: a systematic review and Meta-analysis. JAMA..

[10] Abu Suhaiban H, Grasser LR, Javanbakht A (2019). Mental health of refugees and torture survivors: a critical review of prevalence, predictors, and integrated care. Int J Environ Res Public Health.

[11] Gottvall M, Vaez M, Saboonchi F (2019). Social support attenuates the link between torture exposure and post-traumatic stress disorder among male and female Syrian refugees in Sweden. BMC Int Health Hum Rights.

[12] Amnesty International: Torture. https://www.amnesty.org/en/what-we-do/torture/ (2020). Accessed 4 December 2020.

[13] Morina N, Akhtar A, Barth J, Schnyder U. Psychiatric Disorders in Refugees and Internally Displaced Persons After Forced Displacement: A Systematic Review. Frontiers in psychiatry. 2018;9:433-. 10.3389/fpsyt.2018.00433 PMC6160546 30298022

[14] Morina N, Kuenburg A, Schnyder U, Bryant RA, Nickerson A, Schick M. The Association of Post-traumatic and Postmigration Stress with Pain and Other Somatic Symptoms: An Explorative Analysis in Traumatized Refugees and Asylum Seekers. Pain medicine (Malden, Mass). 2018;19(1):50–9. 10.1093/pm/pnx005 28340069

[15] Rometsch-Ogioun El Sount C, Windthorst P, Denkinger J, Ziser K, Nikendei C, Kindermann D, et al. Chronic pain in refugees with posttraumatic stress disorder (PTSD): A systematic review on patients' characteristics and specific interventions. Journal of psychosomatic research. 2019;118:83–97. 10.1016/j.jpsychores.2018.07.014 30078503

[16] Lies J, Jones L, Ho R (2019). The management of post-traumatic stress disorder and associated pain and sleep disturbance in refugees. BJPsych advances.

[17] Richter K, Baumgaertner L, Niklewski G, Peter L, Koeck M, Kellner S (2020). Sleep disorders in migrants and refugees: a systematic review with implications for personalized medical approach. EPMA J.

[18] Pacella ML, Hruska B, Delahanty DL (2013). The physical health consequences of PTSD and PTSD symptoms: a meta-analytic review. Journal of Anxiety Disorders.

[19] Silove D, Ventevogel P, Rees S (2017). The contemporary refugee crisis: an overview of mental health challenges. World Psychiatry.

[20] Miller KE, Rasmussen A. War exposure, daily stressors, and mental health in conflict and post-conflict settings: Bridging the divide between trauma-focused and psychosocial frameworks. Social science & medicine (1982). 2010;70(1):7–16. 10.1016/j.socscimed.2009.09.029 19854552

[21] Bradby H, Humphris R, Newall D, Phillimore J (2015). Public health aspects of migrant health: a review of the evidence on health status for refugees and asylum seekers in the European region.

[22] Zen AL, Whooley MA, Shoujun Z, Cohen BE (2012). Post-traumatic stress disorder is associated with poor health behaviors: findings from the heart and soul study. Health Psychol.

[23] van Den Berk-Clark C, Secrest S, Walls J, Hallberg E, Lustman PJ, Schneider FD, et al. Association between posttraumatic stress disorder and lack of exercise, poor diet, obesity, and co-occuring smoking: A systematic review and meta-analysis. Health psychology : official journal of the Division of Health Psychology, American Psychological Association. 2018;37(5):407. 10.1037/hea0000593 PMC5922789 29698016

[24] Hall KS, Hoerster KD, Yancy WS (2015). Post-traumatic stress disorder, physical activity, and eating behaviors. Epidemiol Rev.

[25] Assis MAD, Mello MFD, Scorza FA, Cadrobbi MP, Schooedl AF, Silva SGD, et al. Evaluation of physical activity habits in patients with posttraumatic stress disorder. Clinics. 2008;63(4):473–478, DOI: 10.1590/S1807-59322008000400010. 10.1590/S1807-59322008000400010 PMC2664122 18719757

[26] Warburton DE, Nicol CW, Bredin SS (2006). Health benefits of physical activity: the evidence. CMAJ..

[27] Penedo FJ, Dahn JR (2005). Exercise and well-being: a review of mental and physical health benefits associated with physical activity. Curr Opin Psychiatry.

[28] Global Recommendations on Physical Activity for Health. Geneva: World Health Organization (WHO); 2010. https://www.who.int/publications/i/item/9789241599979. Accessed 14 September 2020. 26180873

[29] Rekommendationer om fysisk aktivitet för vuxna. Stockholm: Yrkesföreningar för fysisk aktivitet (YFA), Svenska Läkaresällskapet; 2011. http://www.yfa.se/rekommendationer-for-fysisk-aktivitet/. Accessed 14 September 2020.

[30] Lee IM, Shiroma EJ, Lobelo F, Puska P, Blair SN, Katzmarzyk PT (2012). Effect of physical inactivity on major non-communicable diseases worldwide: an analysis of burden of disease and life expectancy. Lancet.

[31] Kinzie JD, Riley C, McFarland B, Hayes M, Boehnlein J, Leung P, Adams G (2008). High prevalence rates of diabetes and hypertension among refugee psychiatric patients. J Nerv Ment Dis.

[32] Vancampfort D, Richards J, Stubbs B, Akello G, Gbiri CA, Ward PB, Rosenbaum S (2016). Physical activity in people with posttraumatic stress disorder: a systematic review of correlates. J Phys Act Health.

[33] Leardmann CA, Kelton ML, Smith B, Littman AJ, Boyko EJ, Wells TS (2011). Prospectively assessed posttraumatic stress disorder and associated physical activity. Public Health Rep.

[34] Talbot LS, Neylan TC, Metzler TJ, Cohen BE (2014). The mediating effect of sleep quality on the relationship between PTSD and physical activity. J Clin Sleep Med.

[35] Liedl A, Knaevelsrud C (2008). Chronic pain and PTSD: the perpetual avoidance model and its treatment implications. Torture : quarterly journal on rehabilitation of torture victims and prevention of torture..

[36] Rosenbaum S, Vancampfort D, Steel Z, Newby J, Ward PB, Stubbs B (2015). Physical activity in the treatment of post-traumatic stress disorder: a systematic review and meta-analysis. Psychiatry Res.

[37] Oppizzi LM, Umberger R (2018). The effect of physical activity on PTSD. Issues in Mental Health Nursing.

[38] Purgato M, Richards J, Prina E, Kip A, Del Piccolo L, Michencigh G (2021). Efficacy of physical activity interventions on psychological outcomes in refugee, asylum seeker and migrant populations: a systematic review and meta-analysis. Psychol Sport Exerc.

[39] Stewart AL, Hays RD, Wells KB, Rogers WH, Spritzer KL, Greenfield S (1994). Long-term functioning and well-being outcomes associated with physical activity and exercise in patients with chronic conditions in the medical outcomes study. J Clin Epidemiol.

[40] Sjogren Forss K, Mangrio E, Leijon M, Grahn M, Zdravkovic S. Physical Activity in Relation to Wellbeing Among Newly Arrived Refugees in Sweden: A Quantitative Study. Frontiers in public health. 2021;8:532883-. 10.3389/fpubh.2020.532883 PMC7820172 33490005

[41] Vandenbroucke JP, von Elm E, Altman DG, Gøtzsche PC, Mulrow CD, Pocock SJ, et al. Strengthening the Reporting of Observational Studies in Epidemiology (STROBE): explanation and elaboration. PLoS medicine. 2007;4(10):e297-e. 10.1371/journal.pmed.0040297 PMC2020496 17941715

[42] Coleman JK, Ngor PE, Reynolds RK, Quinn EV, Koebnick EC, Young ED (2012). Initial validation of an exercise “vital sign” in electronic medical records. Med Sci Sports Exerc.

[43] Strath SJ, Kaminsky LA, Ainsworth BE, Ekelund U, Freedson PS, Gary RA, Richardson CR, Smith DT, Swartz AM, American Heart Association Physical Activity Committee of the Council on Lifestyle and Cardiometabolic Health and Cardiovascular, Exercise, Cardiac Rehabilitation and Prevention Committee of the Council on Clinical Cardiology, and Council (2013). Guide to the assessment of physical activity: clinical and research applications: a scientific statement from the American Heart Association. Circulation..

[44] Quiles NN, McCullough AK, Piao L. Validity and Reliability of the Exercise Vital Sign Questionnaire in an Ethnically Diverse Group: A Pilot Study. Journal of primary care & community health. 2019;10:2150132719844062-. 10.1177/2150132719844062 PMC6498766 31044638

[45] Mollica FR, Caspi-Yavin FY, Bollini FP, Truong FT, Tor FS, Lavelle FJ (1992). The Harvard trauma questionnaire: validating a cross-cultural instrument for measuring torture, trauma, and posttraumatic stress disorder in Indochinese refugees. J Nerv Ment Dis.

[46] Kip A, Priebe S, Holling H, Morina N (2020). Psychological interventions for posttraumatic stress disorder and depression in refugees: a meta-analysis of randomized controlled trials. Clin Psychol Psychother.

[47] Oruc L, Kapetanovic A, Pojskic N, Miley K, Forstbauer S, Mollica R (2008). Screening for PTSD and depression in Bosnia and Herzegovina: validating the Harvard trauma questionnaire and the Hopkins symptom checklist. Int J Cult Ment Health.

[48] Vindbjerg E, Carlsson J, Mortensen EL, Makransky G, Nielsen T (2020). A Rasch-based validity study of the Harvard trauma questionnaire. J Affect Disord.

[49] Sigvardsdotter E, Nilsson H, Malm A, Tinghög P, Gottvall M, Vaez M, et al. Development and Preliminary Validation of Refugee Trauma History Checklist (RTHC)-A Brief Checklist for Survey Studies. International journal of environmental research and public health. 2017;14(10). 10.3390/ijerph14101175 PMC5664676 28976937

[50] World Health Organization (WHO). Global Health Observatory (GHO) data: Prevalence of insufficient physical activity. 2016. https://www.who.int/data/gho/data/themes/topics/indicator-groups/indicator-group-details/GHO/insufficient-physical-activity. Accessed 18 December 2020.

[51] Guthold R, Stevens GA, Riley LM, Bull FC (2018). Worldwide trends in insufficient physical activity from 2001 to 2016: a pooled analysis of 358 population-based surveys with 1·9 million participants. Lancet Glob Health.

[52] Bauman AEP, Reis RSP, Sallis JFP, Wells JCP, Loos RJFP, Martin BWMD (2012). Correlates of physical activity: why are some people physically active and others not?. The Lancet (British edition).

[53] O’Driscoll T, Banting LK, Borkoles E, Eime R, Polman R (2014). A systematic literature review of sport and physical activity participation in culturally and linguistically diverse (CALD) migrant populations. J Immigr Minor Health.

[54] Sharara E, Akik C, Ghattas H, Makhlouf Obermeyer C. Physical inactivity, gender and culture in Arab countries: a systematic assessment of the literature. 10.1186/s12889-018-5472-z PMC5960209 29776343

[55] Nyboe L, Lund H (2013). Low levels of physical activity in patients with severe mental illness. Nordic Journal of Psychiatry.

[56] Vancampfort D, Firth J, Schuch FB, Rosenbaum S, Mugisha J, Hallgren M, Probst M, Ward PB, Gaughran F, de Hert M, Carvalho AF, Stubbs B (2017). Sedentary behavior and physical activity levels in people with schizophrenia, bipolar disorder and major depressive disorder: a global systematic review and meta-analysis. World Psychiatry.

[57] Shor R, Shalev A (2016). Barriers to involvement in physical activities of persons with mental illness. Health Promot Int.

[58] Spaaij R, Broerse J, Oxford S, Luguetti C, McLachlan F, McDonald B, et al. Sport, Refugees, and Forced Migration: A Critical Review of the Literature. Frontiers in sports and active living. 2019;1:47-. 10.3389/fspor.2019.00047 PMC7739661 33344970

[59] Haith-Cooper M, Waskett C, Montague J, Horne M. Exercise and physical activity in asylum seekers in Northern England; using the theoretical domains framework to identify barriers and facilitators.(Report). BMC Public Health. 2018;18(1). 10.1186/s12889-018-5692-2 PMC6007006 29914467

[60] Global action plan on physical activity 2018–2030: more active people for a healthier world. Geneva: World Health Organization (WHO); 2018. Licence: CC BY-NC-SA 3.0 IGO.

[61] Smith R, Spaaij R, McDonald B (2019). Migrant integration and cultural Capital in the Context of sport and physical activity: a systematic review. J Int Migr Integr.

[62] Ley C, Karus F, Wiesbauer L, Rato Barrio M, Spaaij R. Health, integration and agency: sport participation experiences of asylum seekers. J Refug Stud. 2020. 10.1093/jrs/feaa081.

[63] Nilsson H, Saboonchi F, Gustavsson C, Malm A, Gottvall M. Trauma-afflicted refugees' experiences of participating in physical activity and exercise treatment: a qualitative study based on focus group discussions. European journal of psychotraumatology. 2019;10(1):1699327-. 10.1080/20008198.2019.1699327 PMC6913663 31853335

[64] Füzéki E, Banzer W (2018). Physical activity recommendations for health and beyond in currently inactive populations. Int J Environ Res Public Health.

[65] Warburton DERP, Bredin SSDP (2016). Reflections on physical activity and health: what should we recommend?. Can J Cardiol.

[66] Vancampfort D, Stubbs B, Ward PB, Teasdale S, Rosenbaum S. Why moving more should be promoted for severe mental illness. The Lancet Psychiatry. 2015;2(4):295-. 10.1016/S2215-0366(15)00099-1 26360074

[67] Schuch FB, Stubbs B, Meyer J, Heissel A, Zech P, Vancampfort D, Rosenbaum S, Deenik J, Firth J, Ward PB, Carvalho AF, Hiles SA (2019). Physical activity protects from incident anxiety: a meta-analysis of prospective cohort studies. Depression and anxiety.

[68] Sharp TJ, Harvey AG (2001). Chronic pain and posttraumatic stress disorder: mutual maintenance?. Clin Psychol Rev.

[69] Charuvastra A, Cloitre M (2008). Social bonds and posttraumatic stress disorder. Annu Rev Psychol.

[70] Benight CC, Bandura A (2004). Social cognitive theory of posttraumatic recovery: the role of perceived self-efficacy. Behav Res Ther.

[71] Rutter LA, Weatherill RP, Krill SC, Orazem R, Taft CT (2013). Posttraumatic stress disorder symptoms, depressive symptoms, exercise, and health in college students. Psychol Trauma Theory Res Pract Policy.

[72] Horikoshi N, Iwasa H, Yasumura S, Maeda M (2017). The characteristics of non-respondents and respondents of a mental health survey among evacuees in a disaster: the Fukushima health management survey. Fukushima J Med Sci.

[73] Van Loon AJM, Tijhuis M, Picavet HSJ, Surtees PG, Ormel J (2003). Survey non-response in the Netherlands: effects on prevalence estimates and associations. Ann Epidemiol.

[74] Joanna JJW, Mark B, Louise R (2017). On the impact of nonresponse in logistic regression: application to the 45 and up study. BMC Med Res Methodol.

[75] Golightly YM, Allen KD, Ambrose KR, Stiller JL, Evenson KR, Voisin C, et al. Physical Activity as a Vital Sign: A Systematic Review. Preventing chronic disease. 2017;14:E123-E. 10.5888/pcd14.170030 PMC5716811 29191260

[76] World Medical Association. WMA declaration of Helsinki: Ethical principles for medical research involving human subjects. 2013. https://www.wma.net/policies-post/wma-declaration-of-helsinki-ethical-principles-for-medical-research-involving-human-subjects/. Accessed 16 June 2020. 10.1001/jama.2013.281053 24141714

